# HiJAKing Innate Lymphoid Cells?

**DOI:** 10.3389/fimmu.2017.00438

**Published:** 2017-04-13

**Authors:** Giuseppe Sciumè, Mimi T. Le, Massimo Gadina

**Affiliations:** ^1^Department of Molecular Medicine, Sapienza University of Rome, Laboratory Affiliated to Istituto Pasteur Italia – Fondazione Cenci Bolognetti, Roma, Italy; ^2^Translational Immunology Section, Office of Science Technology (OST), NIAMS, NIH, Bethesda, MD, USA

**Keywords:** innate lymphoid cells, cytokines, Janus kinase (JAK)/signal transducer and activator of transcription (STAT) pathways, autoimmune diseases, JAK inhibitors

The family of innate lymphoid cells (ILCs) consists of a heterogeneous group of cytokine-producing cells that have features in common with adaptive T helper (Th) cells. Cytokines acting through the Janus kinase (JAK)/signal transducer and activator of transcription (STAT) pathways are key players in both Th and ILC biology. Observations in animal models, supported by evidence from humans, have highlighted the importance of the downstream events evoked by the cytokines that signal through the common IL-2 γ-chain receptor. Similarly, it is reasonable to assume that therapeutic targeting of this signaling cascade will also modulate ILC effector function in disease. Since a major limitation of gene knockout studies in mice is the complete loss of ILC populations, including NK cells, we believe that an attractive, alternative, strategy would be to study the role of cytokine signaling in the regulation of ILC function by pharmacological manipulation of these pathways instead. Here, we discuss the potential of JAK inhibitors as a drug class to elucidate mechanisms underlying ILC biology and to inform the design of new therapeutic strategies for inflammatory and autoimmune disorders.

## Distinctive Features of ILC Subsets

In the last 10 years, ILCs have emerged as a new class of lymphocytes with the ability to regulate and amplify the immune response through a variety of effector functions that parallel those of T lymphocytes (1, 2). We now divide ILCs into three groups based on their pattern of cytokine expression (3). NK cells are the only subset with cytolytic ability and, along with other IFN-γ-producing subsets, fall in the group of type 1 ILCs. One major difference between NK cells and other ILC1 is their tissue distribution; while ILC1 are mainly tissue-resident cells, NK cells recirculate in the body (4–7). Type 2 ILCs consist of cells producing IL-13 and IL-5, including nuocytes, natural helper cells, and innate type 2 helper cells. These cells are involved in the resolution of parasitic infections and in allergic airway inflammation (8). The type 3 group comprises cells producing IL-22 and/or IL-17, including lymphoid tissue inducer (LTi)-like cells and a subset expressing NK cell markers (NCR) named NCR^+^ ILC3. Type 3 ILCs are enriched at mucosal sites and contribute in maintaining the integrity at intestinal barriers but also play a key role in promoting inflammation in mouse models of colitis (9). Although a potential role in inflammatory bowel disease can be indirectly inferred in humans, further studies are needed to better understand their function (3, 10).

Generation and development of ILC functional diversity depends on complex network of lineage-defining and signal-dependent transcription factors (LDTFs and SDTFs) that control both ILC and Th cell differentiation (11). The pathways delineating ILC subsets can be simplified according to the requirement of four LDTFs: Eomes for NK cells, T-bet for ILC1, GATA-3 for ILC2, and RORγt for ILC3. However, these TFs also have broad lineage-defining activity and there is considerable overlap among them (12–18). Thus, as for Th cells, the boundaries among ILC lineages are blurred and plasticity across the three groups has been observed both in humans and mice (19–23).

**Figure 1:**
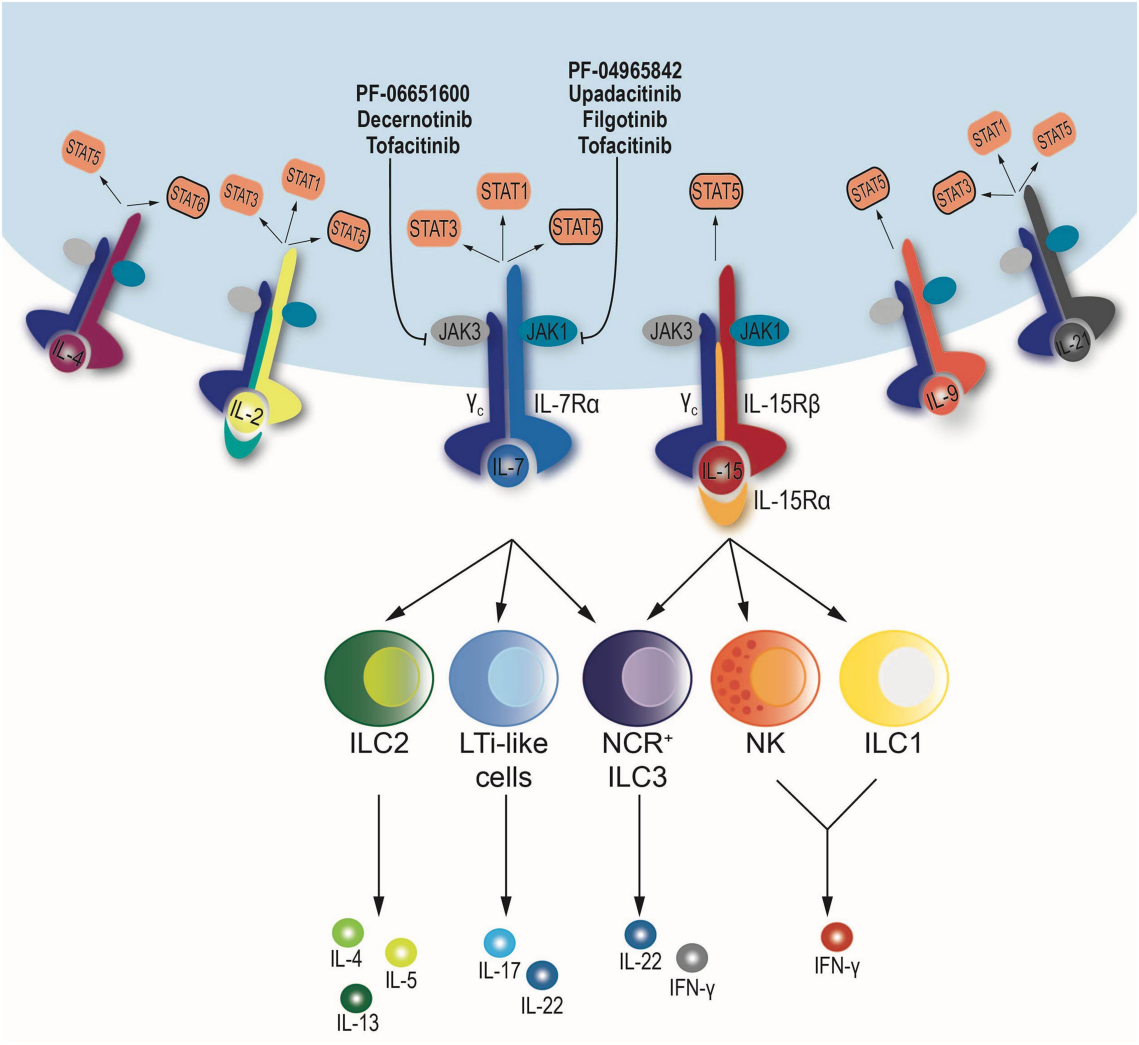
**Schematic diagram of the downstream signaling pathway of cytokines sharing the common γ chain receptor: IL-2, IL-4, IL-7, IL-9, IL-15, and IL-21**. All depend on the same set of Janus kinases (JAKs), JAK1 and JAK3, but may use a different combination signal transducer and activator of transcription (STATs). When cytokines bind to their respective receptors, JAK–STAT phosphorylation occurs. JAK inhibitors blocking JAK1, JAK3, or both are shown. NK cells and ILC1 depend on IL-15, while ILC2 and lymphoid tissue inducer (LTi)-like cells require IL-7 for development and maintenance. Finally, NCR^+^ ILC3 subset relies on IL-7 and IL-15.

One substantial difference between ILCs and T cells is that commitment to ILC lineage is independent of the presence of pathogens and occurs early in development (24). In fact, ILC precursors share epigenetic features with mature ILCs (25, 26). Importantly, a common feature of all ILCs is their requirement for IL-15 and IL-7, two JAK-dependent cytokines that utilize the common γ chain receptor *(IL2RG)* (2). The non-redundant function of these two cytokines makes the JAK/STAT pathway the main signaling cascade involved in ILC development and homeostasis (Figure 1).

## ILC Development and Homeostasis: All Roads Lead to JAK3?

The JAK/STAT pathway transduces signals downstream of type I and type II cytokine receptors and has been described in great details elsewhere (27). Its importance was demonstrated in genetically modified animals and in patients (28). Individuals with mutations of IL-7Rα, IL-2R common γ chain (IL2Rγ), and JAK3 develop severe combined immunodeficiency. Given that these defects are restricted to the immune system, compounds blocking the enzymatic activity of JAKs have been developed as immunosuppressants to be used in immune-mediated diseases. While mutations in IL7Rα cause a T(−), B(+), and NK(+) immunodeficiency, the latter two mutations result in a T(−), B(+), and NK(−). Recently, no ILCs were found in patients with JAK3 and IL-2Rγ mutations (29). Notably, after hematopoietic stem cell transplantation (HSCT) in non-myeloablative conditioning regimens, patients remained ILC(−), while ILCs were partially reconstituted only in myeloablative HSCT. Altogether, these findings highlight the importance of common γ chain cytokines on the development of T, NK cells, and ILCs.

The non-redundant role of IL-15 in the regulation of NK cell differentiation and homeostasis has long been appreciated (30–33). Recently, a critical role for IL-15 has been also shown for several subsets of tissue-resident ILC1 (34–36). Conversely, IL-7 is required for ILC2 and ILC3 development and maintenance (37, 38). Nonetheless, not all the subsets conform to this dualistic model. For instance, NCR^+^ ILC3 require IL-7 as do other ILC3 subsets, but IL-15 also affects their maintenance (35, 38). Moreover, IL-7Rα-deficient mice show a more severe defect in ILC2 and LTi-like ILC3 numbers as compared to IL-7-deficient mice, probably because of the cytokine TSLP, which also signals through the IL-7Rα (39, 40). Finally, T cell-derived IL-2 also regulates the number of NK cells, and this effect becomes evident in the absence of regulatory T cells (41). Overall, among the signaling molecules downstream of all these cytokines, JAK3 and JAK1 appear to have a critical role as gatekeeper of the signals leading to activation of SDTF like the STATs.

## ILCs: Facts and STATs

It is not surprising that STATs have a major role in ILC functions since they transduce signals received by cytokine–cytokine receptor interactions. For example, the role of STAT5 in NK cells has been investigated using several mouse models that show the key role of this TF in the biology of NK cells (42–45). However, in terms of lineage diversification, the requirement of STATs between ILCs and Th cells does not overlap.

The traditional “monolithic” view of Th differentiation relies on the paradigm “one STAT/one subset.” In this model, STAT4 is the main driver of Th1 development, STAT6 is critical for Th2, and STAT3 for Th17/22 (46). Although Th differentiation is now thought to be a fluid process based on networks of TFs, activation of selected STATs is still thought to drive the generation of distinct Th subsets. In contrast, ILC diversification is not driven by selective activation of STATs. Notably, several studies have shown no role for STAT4 in the regulation of type 1 ILC differentiation, STAT6 for ILC2 nor STAT3 for ILC3 (6, 47, 48). However, activation of distinct STATs is important for ILC function: deficiency of STAT4 profoundly affects NK cell and ILC1 responses during infections. Similarly, STAT6-deficient ILC2 produce less IL-13, while STAT3 controls production of IL-22 in ILC3 (6, 18, 47–50). Thus, the paradigm “one STAT/one subset” better reflects the effector functions of distinct ILCs, whereas lineage diversification is probably obtained through early expression of LDTFs, also known as the “master regulators.” What regulates the regulators is still unknown, but the JAK/STAT pathway represents an obvious candidate and could be modulated during ILC activation and alter their effector function.

## Targeting JAKs in ILCs

Given the critical role of IL-2Rγ-using cytokines for ILCs, targeting their signaling cascade could be used to modulate ILC function. The non-selective JAK inhibitor, tofacitinib, is currently approved for the treatment of rheumatoid arthritis. In this context, *ex vivo* treatment of CD4^+^ T cells with tofacitinib affects the differentiation programs of Th cells (51) and alters the expression of rheumatoid arthritis risk genes endowed with super enhancer structure (52). Tofacitinib and other “first generation” JAK inhibitors block multiple JAKs and, therefore, inhibit the actions of a large variety of cytokines. Several JAK-selective inhibitors are being developed. Molecules like decernotinib and PF-06651600 (JAK3 selective) are already in late-phase clinical development, but they are also useful tools to understand the biological role of JAK3 in ILCs. On the other hand, given that several of the cytokines mentioned above also signal through JAK1, compounds like filgotinib (JAK1 selective but with some activity on JAK2), upadacitinib, and PF-04965842 (JAK1 selective) could be very helpful to understand the biological role of each the JAKs (53). Interestingly, tofacitinib has shown promising results in the treatment of ulcerative colitis but a lack of efficacy in Crohn’s disease whereas filgotinib has shown some efficacy. Given the role that ILCs have in the gastrointestinal immune response, we are tempted to speculate that altering the effector functions of ILCs could contribute to these different responses.

In homeostatic condition, mice treated with JAK inhibitors show no major changes in the pool of adaptive immune cells, with the only exception being FoxP3^+^ regulatory T cells, which decrease following JAK1 inhibition (54). On the other hand, JAK3 and JAK1 inhibition significantly decreased the frequency of NK cells (54–56). At the transcriptional level, NK cell effector programs are similarly affected by both JAK1 and JAK3 inhibition (54). Similar results have been obtained using NK cells treated *ex vivo* with IL-2, where activation of several target genes is inhibited by targeting both JAK1 and JAK3. From a therapeutic point of view, it is interesting to note that a JAK1-selective inhibitor is more effective than a JAK3-selective inhibitor in blocking secondary autocrine responses induced by IFN-γ released by activated NK cells (54).

## Conclusion

Innate lymphoid cells are now recognized as critical components of the immune response and translational studies have shown that they also play a role in immune-mediated diseases (57–59). Like Th cells, ILCs are dependent on specific cytokines signaling through JAK1 and JAK3 for their development and acquisition of effector function. Single gene knockout animals have limited use in the study of ILCs as they cause significant perturbation of immune compartments. We suggest that the availability of drugs that specifically block the JAK/STAT pathway can be very useful in the study of ILCs and may, to an extent, obviate the need for gene knockout animals in the study of ILC biology. Furthermore, JAK inhibitors are already in clinical use, so the effect of these drugs on ILCs in patients being treated in the clinic for autoimmune and inflammatory diseases will also shortly be evident.

## Author Contributions

GS and MG wrote the manuscript. ML designed the figure, wrote the manuscript, and made the necessary edits. The final manuscript was a result of the joint efforts of all the authors.

## Conflict of Interest Statement

The authors declare that the research was conducted in the absence of any commercial or financial relationships that could be construed as a potential conflict of interest.

## References

[1] Eberl G Colonna M Di Santo JP McKenzie AN. Innate lymphoid cells. Innate lymphoid cells: a new paradigm in immunology. Science (2015) 348(6237):aaa6566. 10.1126/science.aaa6566 25999512 PMC5658207

[2] Spits H Di Santo JP. The expanding family of innate lymphoid cells: regulators and effectors of immunity and tissue remodeling. Nat Immunol (2011) 12(1):21–7. 10.1038/ni.1962 21113163

[3] Spits H Artis D Colonna M Diefenbach A Di Santo JP Eberl G Innate lymphoid cells – a proposal for uniform nomenclature. Nat Rev Immunol (2013) 13(2):145–9. 10.1038/nri3365 23348417

[4] Peng H Jiang X Chen Y Sojka DK Wei H Gao X Liver-resident NK cells confer adaptive immunity in skin-contact inflammation. J Clin Invest (2013) 123(4):1444–56. 10.1172/JCI66381 23524967 PMC3613925

[5] Gasteiger G Fan X Dikiy S Lee SY Rudensky AY. Tissue residency of innate lymphoid cells in lymphoid and nonlymphoid organs. Science (2015) 350(6263):981–5. 10.1126/science.aac9593 26472762 PMC4720139

[6] O’Sullivan TE Rapp M Fan X Weizman OE Bhardwaj P Adams NM Adipose-resident group 1 innate lymphoid cells promote obesity-associated insulin resistance. Immunity (2016) 45(2):428–41. 10.1016/j.immuni.2016.06.016 27496734 PMC5004886

[7] Bernardini G Sciume G Santoni A. Differential chemotactic receptor requirements for NK cell subset trafficking into bone marrow. Front Immunol (2013) 4:12. 10.3389/fimmu.2013.00012 23386850 PMC3558687

[8] Morita H Moro K Koyasu S. Innate lymphoid cells in allergic and nonallergic inflammation. J Allergy Clin Immunol (2016) 138(5):1253–64. 10.1016/j.jaci.2016.09.011 27817797

[9] Song C Lee JS Gilfillan S Robinette ML Newberry RD Stappenbeck TS Unique and redundant functions of NKp46+ ILC3s in models of intestinal inflammation. J Exp Med (2015) 212(11):1869–82. 10.1084/jem.20151403 26458769 PMC4612098

[10] Forkel M Mjosberg J. Dysregulation of group 3 innate lymphoid cells in the pathogenesis of inflammatory bowel disease. Curr Allergy Asthma Rep (2016) 16(10):73. 10.1007/s11882-016-0652-3 27645534 PMC5028403

[11] Shih HY Sciume G Poholek AC Vahedi G Hirahara K Villarino AV Transcriptional and epigenetic networks of helper T and innate lymphoid cells. Immunol Rev (2014) 261(1):23–49. 10.1111/imr.12208 25123275 PMC4321863

[12] Yagi R Zhong C Northrup DL Yu F Bouladoux N Spencer S The transcription factor GATA3 is critical for the development of all IL-7Ralpha-expressing innate lymphoid cells. Immunity (2014) 40(3):378–88. 10.1016/j.immuni.2014.01.012 24631153 PMC4026797

[13] Serafini N Klein Wolterink RG Satoh-Takayama N Xu W Vosshenrich CA Hendriks RW GATA3 drives development of RORgammat+ group 3 innate lymphoid cells. J Exp Med (2014) 211(2):199–208. 10.1084/jem.20131038 24419270 PMC3920560

[14] Zhong C Cui K Wilhelm C Hu G Mao K Belkaid Y Group 3 innate lymphoid cells continuously require the transcription factor GATA-3 after commitment. Nat Immunol (2016) 17(2):169–78. 10.1038/ni.3318 26595886 PMC4718889

[15] Sciume G Hirahara K Takahashi H Laurence A Villarino AV Singleton KL Distinct requirements for T-bet in gut innate lymphoid cells. J Exp Med (2012) 209(13):2331–8. 10.1084/jem.20122097 23209316 PMC3526352

[16] Klose CS Kiss EA Schwierzeck V Ebert K Hoyler T d’Hargues Y A T-bet gradient controls the fate and function of CCR6-RORgammat+ innate lymphoid cells. Nature (2013) 494(7436):261–5. 10.1038/nature11813 23334414

[17] Rankin LC Groom JR Chopin M Herold MJ Walker JA Mielke LA The transcription factor T-bet is essential for the development of NKp46+ innate lymphocytes via the Notch pathway. Nat Immunol (2013) 14(4):389–95. 10.1038/ni.2545 23455676 PMC4076532

[18] Rankin LC Girard-Madoux MJ Seillet C Mielke LA Kerdiles Y Fenis A Complementarity and redundancy of IL-22-producing innate lymphoid cells. Nat Immunol (2016) 17(2):179–86. 10.1038/ni.3332 26595889 PMC4720992

[19] Cella M Otero K Colonna M. Expansion of human NK-22 cells with IL-7, IL-2, and IL-1beta reveals intrinsic functional plasticity. Proc Natl Acad Sci U S A (2010) 107(24):10961–6. 10.1073/pnas.1005641107 20534450 PMC2890739

[20] Bernink JH Krabbendam L Germar K de Jong E Gronke K Kofoed-Nielsen M Interleukin-12 and -23 control plasticity of CD127(+) group 1 and group 3 innate lymphoid cells in the intestinal lamina propria. Immunity (2015) 43(1):146–60. 10.1016/j.immuni.2015.06.019 26187413

[21] Ohne Y Silver JS Thompson-Snipes L Collet MA Blanck JP Cantarel BL IL-1 is a critical regulator of group 2 innate lymphoid cell function and plasticity. Nat Immunol (2016) 17(6):646–55. 10.1038/ni.3447 27111142

[22] Silver JS Kearley J Copenhaver AM Sanden C Mori M Yu L Inflammatory triggers associated with exacerbations of COPD orchestrate plasticity of group 2 innate lymphoid cells in the lungs. Nat Immunol (2016) 17(6):626–35. 10.1038/ni.3443 27111143 PMC5345745

[23] Huang Y Guo L Qiu J Chen X Hu-Li J Siebenlist U IL-25-responsive, lineage-negative KLRG1(hi) cells are multipotential ’inflammatory’ type 2 innate lymphoid cells. Nat Immunol (2015) 16(2):161–9. 10.1038/ni.3078 25531830 PMC4297567

[24] Gronke K Kofoed-Nielsen M Diefenbach A Innate lymphoid cells, precursors and plasticity. Immunol Lett (2016) 179:9–18. 10.1016/j.imlet.2016.07.004 27394700

[25] Shih HY Sciume G Mikami Y Guo L Sun HW Brooks SR Developmental acquisition of regulomes underlies innate lymphoid cell functionality. Cell (2016) 165(5):1120–33. 10.1016/j.cell.2016.04.029 27156451 PMC4874839

[26] Lim AI Li Y Lopez-Lastra S Stadhouders R Paul F Casrouge A Systemic human ILC precursors provide a substrate for tissue ILC differentiation. Cell (2017) 168(6):1086–100.e1010. 10.1016/j.cell.2017.02.021 28283063

[27] O’Shea JJ Murray PJ. Cytokine signaling modules in inflammatory responses. Immunity (2008) 28(4):477–87. 10.1016/j.immuni.2008.03.002 18400190 PMC2782488

[28] O’Shea JJ Plenge R. JAK and STAT signaling molecules in immunoregulation and immune-mediated disease. Immunity (2012) 36(4):542–50. 10.1016/j.immuni.2012.03.014 22520847 PMC3499974

[29] Vely F Barlogis V Vallentin B Neven B Piperoglou C Ebbo M Evidence of innate lymphoid cell redundancy in humans. Nat Immunol (2016) 17(11):1291–9. 10.1038/ni.3553 27618553 PMC5074366

[30] Ranson T Vosshenrich CA Corcuff E Richard O Muller W Di Santo JP. IL-15 is an essential mediator of peripheral NK-cell homeostasis. Blood (2003) 101(12):4887–93. 10.1182/blood-2002-11-3392 12586624

[31] Vosshenrich CA Ranson T Samson SI Corcuff E Colucci F Rosmaraki EE Roles for common cytokine receptor gamma-chain-dependent cytokines in the generation, differentiation, and maturation of NK cell precursors and peripheral NK cells in vivo. J Immunol (2005) 174(3):1213–21. 10.4049/jimmunol.174.3.1213 15661875

[32] Kennedy MK Glaccum M Brown SN Butz EA Viney JL Embers M Reversible defects in natural killer and memory CD8 T cell lineages in interleukin 15-deficient mice. J Exp Med (2000) 191(5):771–80. 10.1084/jem.191.5.771 10704459 PMC2195858

[33] Huntington ND. The unconventional expression of IL-15 and its role in NK cell homeostasis. Immunol Cell Biol (2014) 92(3):210–3. 10.1038/icb.2014.1 24492800

[34] Satoh-Takayama N Vosshenrich CA Lesjean-Pottier S Sawa S Lochner M Rattis F Microbial flora drives interleukin 22 production in intestinal NKp46+ cells that provide innate mucosal immune defense. Immunity (2008) 29(6):958–70. 10.1016/j.immuni.2008.11.001 19084435

[35] Klose CS Flach M Mohle L Rogell L Hoyler T Ebert K Differentiation of type 1 ILCs from a common progenitor to all helper-like innate lymphoid cell lineages. Cell (2014) 157(2):340–56. 10.1016/j.cell.2014.03.030 24725403

[36] Cortez VS Fuchs A Cella M Gilfillan S Colonna M. Cutting edge: salivary gland NK cells develop independently of Nfil3 in steady-state. J Immunol (2014) 192(10):4487–91. 10.4049/jimmunol.1303469 24740507

[37] Moro K Yamada T Tanabe M Takeuchi T Ikawa T Kawamoto H Innate production of T(H)2 cytokines by adipose tissue-associated c-Kit(+)Sca-1(+) lymphoid cells. Nature (2010) 463(7280):540–4. 10.1038/nature08636 20023630

[38] Satoh-Takayama N Lesjean-Pottier S Vieira P Sawa S Eberl G Vosshenrich CA IL-7 and IL-15 independently program the differentiation of intestinal CD3-NKp46+ cell subsets from Id2-dependent precursors. J Exp Med (2010) 207(2):273–80. 10.1084/jem.20092029 20142427 PMC2822619

[39] Vonarbourg C Mortha A Bui VL Hernandez PP Kiss EA Hoyler T Regulated expression of nuclear receptor RORgammat confers distinct functional fates to NK cell receptor-expressing RORgammat(+) innate lymphocytes. Immunity (2010) 33(5):736–51. 10.1016/j.immuni.2010.10.017 21093318 PMC3042726

[40] Hoyler T Klose CS Souabni A Turqueti-Neves A Pfeifer D Rawlins EL The transcription factor GATA-3 controls cell fate and maintenance of type 2 innate lymphoid cells. Immunity (2012) 37(4):634–48. 10.1016/j.immuni.2012.06.020 23063333 PMC3662874

[41] Gasteiger G Hemmers S Bos PD Sun JC Rudensky AY. IL-2-dependent adaptive control of NK cell homeostasis. J Exp Med (2013) 210(6):1179–87. 10.1084/jem.20122571 23650439 PMC3674698

[42] Yao Z Cui Y Watford WT Bream JH Yamaoka K Hissong BD Stat5a/b are essential for normal lymphoid development and differentiation. Proc Natl Acad Sci U S A (2006) 103(4):1000–5. 10.1073/pnas.0507350103 16418296 PMC1327727

[43] Eckelhart E Warsch W Zebedin E Simma O Stoiber D Kolbe T A novel Ncr1-Cre mouse reveals the essential role of STAT5 for NK-cell survival and development. Blood (2011) 117(5):1565–73. 10.1182/blood-2010-06-291633 21127177

[44] Imada K Bloom ET Nakajima H Horvath-Arcidiacono JA Udy GB Davey HW Stat5b is essential for natural killer cell-mediated proliferation and cytolytic activity. J Exp Med (1998) 188(11):2067–74. 10.1084/jem.188.11.2067 9841920 PMC2212377

[45] Lin JX Li P Liu D Jin HT He J Ata Ur Rasheed M Critical role of STAT5 transcription factor tetramerization for cytokine responses and normal immune function. Immunity (2012) 36(4):586–99. 10.1016/j.immuni.2012.02.017 22520852 PMC3551341

[46] O’Shea JJ Paul WE Mechanisms underlying lineage commitment and plasticity of helper CD4+ T cells. Science (2010) 327(5969):1098–102. 10.1126/science.1178334 20185720 PMC2997673

[47] Nussbaum JC Van Dyken SJ von Moltke J Cheng LE Mohapatra A Molofsky AB Type 2 innate lymphoid cells control eosinophil homeostasis. Nature (2013) 502(7470):245–8. 10.1038/nature12526 24037376 PMC3795960

[48] Guo X Qiu J Tu T Yang X Deng L Anders RA Induction of innate lymphoid cell-derived interleukin-22 by the transcription factor STAT3 mediates protection against intestinal infection. Immunity (2014) 40(1):25–39. 10.1016/j.immuni.2013.10.021 24412612 PMC3919552

[49] Sun JC Madera S Bezman NA Beilke JN Kaplan MH Lanier LL. Proinflammatory cytokine signaling required for the generation of natural killer cell memory. J Exp Med (2012) 209(5):947–54. 10.1084/jem.20111760 22493516 PMC3348098

[50] Miyagi T Gil MP Wang X Louten J Chu WM Biron CA. High basal STAT4 balanced by STAT1 induction to control type 1 interferon effects in natural killer cells. J Exp Med (2007) 204(10):2383–96. 10.1084/jem.20070401 17846149 PMC2118450

[51] Ghoreschi K Jesson MI Li X Lee JL Ghosh S Alsup JW Modulation of innate and adaptive immune responses by tofacitinib (CP-690,550). J Immunol (2011) 186(7):4234–43. 10.4049/jimmunol.1003668 21383241 PMC3108067

[52] Vahedi G Kanno Y Furumoto Y Jiang K Parker SC Erdos MR Super-enhancers delineate disease-associated regulatory nodes in T cells. Nature (2015) 520(7548):558–62. 10.1038/nature14154 25686607 PMC4409450

[53] Telliez JB Dowty ME Wang L Jussif J Lin T Li L Discovery of a JAK3-selective inhibitor: functional differentiation of JAK3-selective inhibition over pan-JAK or JAK1-selective inhibition. ACS Chem Biol (2016) 11(12):3442–51. 10.1021/acschembio.6b00677 27791347

[54] Moodley D Yoshida H Mostafavi S Asinovski N Ortiz-Lopez A Symanowicz P Network pharmacology of JAK inhibitors. Proc Natl Acad Sci U S A (2016) 113(35):9852–7. 10.1073/pnas.1610253113 27516546 PMC5024632

[55] Schonberg K Rudolph J Vonnahme M Parampalli Yajnanarayana S Cornez I Hejazi M JAK inhibition impairs NK cell function in myeloproliferative neoplasms. Cancer Res (2015) 75(11):2187–99. 10.1158/0008-5472.CAN-14-3198 25832652

[56] Valenzuela F Papp KA Pariser D Tyring SK Wolk R Buonanno M Effects of tofacitinib on lymphocyte sub-populations, CMV and EBV viral load in patients with plaque psoriasis. BMC Dermatol (2015) 15:8. 10.1186/s12895-015-0025-y 25951857 PMC4436155

[57] Wohlfahrt T Usherenko S Englbrecht M Dees C Weber S Beyer C Type 2 innate lymphoid cell counts are increased in patients with systemic sclerosis and correlate with the extent of fibrosis. Ann Rheum Dis (2016) 75(3):623–6. 10.1136/annrheumdis-2015-207388 26338035

[58] Cols M Rahman A Maglione PJ Garcia-Carmona Y Simchoni N Ko HB Expansion of inflammatory innate lymphoid cells in patients with common variable immune deficiency. J Allergy Clin Immunol (2016) 137(4):1206–15.e1201–6. 10.1016/j.jaci.2015.09.013 26542033 PMC4866594

[59] Roan F Stoklasek TA Whalen E Molitor JA Bluestone JA Buckner JH CD4+ group 1 innate lymphoid cells (ILC) form a functionally distinct ILC subset that is increased in systemic sclerosis. J Immunol (2016) 196(5):2051–62. 10.4049/jimmunol.1501491 26826243 PMC4761490

